# Pharmacometabolomic Signature of Ataxia SCA1 Mouse Model and Lithium Effects

**DOI:** 10.1371/journal.pone.0070610

**Published:** 2013-08-02

**Authors:** Bertrand Perroud, Paymaan Jafar-Nejad, William R. Wikoff, Jennifer R. Gatchel, Lu Wang, Dinesh K. Barupal, Juan Crespo-Barreto, Oliver Fiehn, Huda Y. Zoghbi, Rima Kaddurah-Daouk

**Affiliations:** 1 UC Davis Genome Center, University of California Davis, Davis, California, United States of America; 2 Jan and Dan Duncan Neurological Research Institute at Texas Children's Hospital, Department of Molecular and Human Genetics, Baylor College of Medicine, Houston, Texas, United States of America; 3 Department of Biostatistics, School of Public Health, University of California Los Angeles, Los Angeles, California, United States of America; 4 The Departments of Pediatrics, Neurology, and Neuroscience and the Howard Hughes Medical Institute, Baylor College of Medicine, Houston, Texas, United States of America; 5 Department of Psychiatry and Behavioral Sciences, Duke University Medical Center, Durham, North Carolina, United States of America; University College London, United Kingdom

## Abstract

We have shown that lithium treatment improves motor coordination in a spinocerebellar ataxia type 1 (SCA1) disease mouse model (*Sca1^154Q/+^*). To learn more about disease pathogenesis and molecular contributions to the neuroprotective effects of lithium, we investigated metabolomic profiles of cerebellar tissue and plasma from SCA1-model treated and untreated mice. Metabolomic analyses of wild-type and *Sca1^154Q/+^* mice, with and without lithium treatment, were performed using gas chromatography time-of-flight mass spectrometry and BinBase mass spectral annotations. We detected 416 metabolites, of which 130 were identified. We observed specific metabolic perturbations in *Sca1^154Q/+^* mice and major effects of lithium on metabolism, centrally and peripherally. Compared to wild-type, *Sca1^154Q/+^* cerebella metabolic profile revealed changes in glucose, lipids, and metabolites of the tricarboxylic acid cycle and purines. Fewer metabolic differences were noted in *Sca1^154Q/+^* mouse plasma versus wild-type. In both genotypes, the major lithium responses in cerebellum involved energy metabolism, purines, unsaturated free fatty acids, and aromatic and sulphur-containing amino acids. The largest metabolic difference with lithium was a 10-fold increase in ascorbate levels in wild-type cerebella (p<0.002), with lower threonate levels, a major ascorbate catabolite. In contrast, *Sca1^154Q/+^* mice that received lithium showed no elevated cerebellar ascorbate levels. Our data emphasize that lithium regulates a variety of metabolic pathways, including purine, oxidative stress and energy production pathways. The purine metabolite level, reduced in the *Sca1^154Q/+^* mice and restored upon lithium treatment, might relate to lithium neuroprotective properties.

## Introduction

Spinocerebellar ataxia type 1 (SCA1) is an autosomal dominant neurodegenerative disease that is caused by the expansion of a translated CAG repeat in *ATAXIN1* (*ATXN1*). SCA1 is characterized by progressive loss of balance and coordination, mild cognitive impairments, speaking and swallowing difficulties, and eventually respiratory failure leading to premature death [1]. The toxic effects of the glutamine-expanded protein result in variable degrees of neurodegeneration, predominantly in the cerebellum, brainstem and spinocerebellar tracts [2]. A knock-in mouse model of SCA1 (*Sca1^154Q/+^*) recapitulates many aspects of the human disease [3], enabling us to study SCA1 pathophysiology and test therapeutic candidates. Molecular mechanisms that underlie the pathophysiology of SCA1 are slowly becoming understood [4]. The dysregulation of several neuronal genes that has been observed in the tissue of humans with SCA1 has also been found in the Purkinje cells of SCA1 transgenic mice, even at the pre-symptomatic stage [4]. In light of this notion, Watase et al. [5] treated *Sca1^154Q/+^* mice with lithium—which exerts neuroprotective effects possibly by affecting gene transcription—and demonstrated that lithium rescues several SCA1 phenotypes in this model.

Lithium has been the standard pharmacological treatment for bipolar disorder for over fifty years. During the past two decades, attention has been drawn to the neuroprotective properties of lithium against diverse insults, including glutamate-induced excitotoxicity and endoplasmic reticulum stress [6]. Multiple molecular pathways such as phosphoinositides [7], [8], protein kinase C signaling pathway [9] and glycogen synthase kinase 3 activity could explain some of the neuroprotective properties of lithium [10]–[13].

It is unclear how lithium improves the SCA1 disease model and what metabolic changes might be modified. There is no biological marker for following the SCA1 disease course or for measuring the effects of lithium or any other therapy on patients under the treatment. To seek potential markers and a deeper understanding of the disease pathogenesis and response to lithium, we applied techniques from the rapidly-evolving field of metabolomics, which enables the identification and quantification of hundreds of small molecules in cells, tissues and body fluids. The metabolic profile of an individual captures a metabolic state at a certain point in time and is regulated by net interactions between gene products and environmental influences [14]. Metabolic profiles could provide valuable insights into disease mechanisms, and can lead to the development of diagnostic and intervention assessment markers [15]–[17]. Metabolic signatures have been found for a variety of diseases, including neurodegenerative disorders [15], [17], substance abuse [18], [19] and drugs used for the treatment of these disorders [15], [20].

Differences between individuals make it difficult to find a homogeneous group of human participants for studying the final metabolic output of the treatment and disease. Having an authentic mouse model of SCA1 disease [3] together with a carefully controlled lithium treatment provides the opportunity to discover biochemical changes in SCA1 disease and lithium treatment which can then be specifically explored in human patients.

In this study, we used a non-targeted mass-spectrometry-based metabolomics platform to map biochemical changes in the cerebellum and plasma of *Sca1^154Q/+^* mice with and without lithium treatment compared to their wild-type littermates.

## Materials and Methods

### Mouse maintenance, diet and dissection

Mouse generation and treatment were performed as described in Watase et al. [5]. In brief, *Sca1^154Q/+^* mice and their wild-type littermates were obtained from crossings between male *Sca1^154Q/+^* mice and wild-type female mice. All mice were on pure C57Bl/6 background. The mice were randomly assigned to two groups at the time of weaning (presymptomatic), when treatment was initiated. Animals were housed in groups of 3 to 5 per cage in a 12 hour light/dark cycle, with free access to food and water. For 10–12 weeks, each group was fed either chow containing 0.2% lithium carbonate or control Purina rodent chow (Harlan Teklad, http://www.teklad.com). At 13–15 weeks of age, the animals were anesthetized. Their blood was obtained via cardiac puncture and collected in EDTA-containing separator tubes. The plasma was separated from blood cells, aliquoted and frozen. The cerebella were dissected on a chilled plate and frozen in liquid nitrogen. All samples were kept at −80°C for later analysis. Mouse experiments followed protocols approved by the Baylor College of Medicine Institutional Animal Care and Use Committee and the Institutional Animal Care and Use Committee of Tokyo Medical and Dental University.

### Blood sample preparation analysis

Plasma samples (30 µl) were thawed on ice and vortexed for 10 seconds. Aliquots (15 µl) were extracted with 1 ml of a carefully degassed solvent mixture of 3∶3∶2 (v/v/v) acetonitrile∶isopropanol∶water at −20°C to perform protein precipitation and metabolite extraction. The plasma/solvent mixture was vortexed for 10 seconds and shaken for 4–6 minutes at 4°C. A subsequent centrifugation step removed insoluble proteins and cell membrane components. The supernatant was dried down using a speed vacuum concentration system (Labconco Centrivap cold trap). To remove most of the membrane lipids and triglycerides, which may interfere with the analysis of amino acids in gas chromatograph mass spectroscopy, 500 µl of a degassed mixture of 1∶1 (v/v) acetonitrile/water was added and the sample underwent vortexing for 10 seconds, ultrasonication for 20 seconds and centrifugation at 13,000× *g* for 2 minutes. Afterward, 450 µl were decanted, which was dried down by speed vac for derivatization and analysis. A mixture of internal retention index markers was prepared using fatty acid methyl esters of C8, C9, C10, C12, C14, C16, C18, C20, C22, C24, C26, C28 and C30 linear chain length, and was dissolved in chloroform at a concentration of 0.8 mg/ml (C8–C16) and 0.4 mg/ml (C18–C30). One µl of this retention index mixture was added to the dried extracts. Ten µl of a solution of 20 mg/ml of 98% pure methoxyamine hydrochloride (Sigma, St. Louis MO) in pyridine (silylation grade, Pierce, Rockford IL) was added and shaken at 30°C for 90 minutes to protect aldehyde and ketone groups. Ninety µl of N-methy-N-(trimethylsilyl) trifluoroacetamide (1 ml bottles, Sigma-Aldrich) was added for trimethylsilylation of acidic protons and shaken at 37°C for 30 minutes. The reaction mixture was transferred to a 2 ml clear glass autosampler vial with microinsert (Agilent, Santa Clara CA) and closed using an 11 mm T/S/T crimp cap (MicroLiter, Suwanee GA).

### Gas Chromatograph Time-of-Flight mass spectrometry

A Gerstel MPS2 automatic liner exchange system was used to inject 0.5 µl of sample at 50°C (ramped to 250°C) in splitless mode with 25 seconds splitless time. An Agilent 6890 gas chromatograph (Santa Clara CA) was used with a 30 m long, 0.25 mm i.d. Rtx5Sil-MS column with 0.25 µm 5% diphenyl film and an additional 10 m integrated guard column (Restek, Bellefonte PA). Chromatography was performed at a constant flow of 1 ml/minute, ramping the oven temperature from 50°C to 330°C with 22 minutes total run time. Mass spectrometry was performed using a Leco Pegasus IV time-of-flight mass spectrometer with a 280°C transfer line temperature, electron ionization at −70 V and an ion source temperature of 250°C. Mass spectra were acquired from m/z 85–500 at 17 spectra s-1 and 1850 V detector voltage.

Result files were exported to the Fiehnlab servers and further processed by the in-house metabolomics BinBase database. All database entries in BinBase were matched against the Fiehn mass spectral library of 1,200 authentic metabolite spectra using retention index and mass spectrum information or the National Institute of Standards and Technology, version 5 commercial library. Identified metabolites were reported if they were present in at least 50% of the samples per study design group (as defined in the SetupX database). Quantitative data were normalized to the sum intensities of all known metabolites and used for statistical investigation.

### Statistical analysis

Multivariate statistics and one-way analysis of variance (ANOVA) models were calculated using Statistica data miner V8 (Statsoft, Tulsa OK, USA). The ANOVA results were used for box-whisker plots and tables, and were subsequently converted into a Cytoscape node attribute file which was utilized to visualize the differential statistics on network graphs. Multivariate partial least square analysis was performed using the non-linear iterative partial lease squares algorithm using unit standard deviation scaling, 50 iterations with 0.0001 as convergence criterion and 7-fold cross validation of the resulting partial least square (PLS) models.

### Network representation

Molfile-encoded chemical structures were retrieved from the PubChem database. A pair-wise similarity matrix of Tanimoto similarity coefficients among all the structures was performed using the online structural clustering tool hosted at the PubChem Web site. The matrix was subsequently converted into a Cytoscape simple interaction format (SIF) formatted network using an in-house Javascript (script available upon request). Kyoto Encyclopedia of Genes and Genomes (KEGG) reaction pair relationships were downloaded from the KEGG ftp site and parsed for the main reactant pairs information. Using MS Excel, a single-step reaction network was constructed for the identified metabolites and saved as a SIF-formatted network. Both networks were imported into Cytoscape [21] and merged using the ‘merge network’ plug-in. Network graphs were visualized using an organic layout. Results of differential ANOVA statistics and relative abundance changes were mapped onto the node size (for magnitude of change), node color (for up/downregulation) and ANOVA p-value (color intensity), respectively.

## Results

The overall study design has eight classes (2 genotypes×2 treatments×2 tissues) as shown in Table 1. Four hundred and sixteen metabolites (416) were quantified, of which we identified 130 non-redundant identified compounds for analysis of impact on metabolic networks. Structural identification of the 286 yet-unidentified markers is a time-consuming and costly process and thus beyond the scope of the work presented here. Raw data are downloadable at http://fiehnlab.ucdavis.edu:8080/m1/main_public.jsp [22]–[24] and attached as processed result files with quantification ions, full mass spectra, retention indices, database identifiers, names and external database references.

**Table 1:** Overall study design with the number of tested animals.

Test Group	Plasma		Cerebellum	
	Wild-Type	*Sca1^154Q/+^*	Wild-Type	*Sca1^154Q/+^*
Control	11	16	10	16
Lithium treated	12	18	11	18

### Plasma and cerebellar metabolic signature for Sca1^154Q/+^ mice

**Figure 1:**
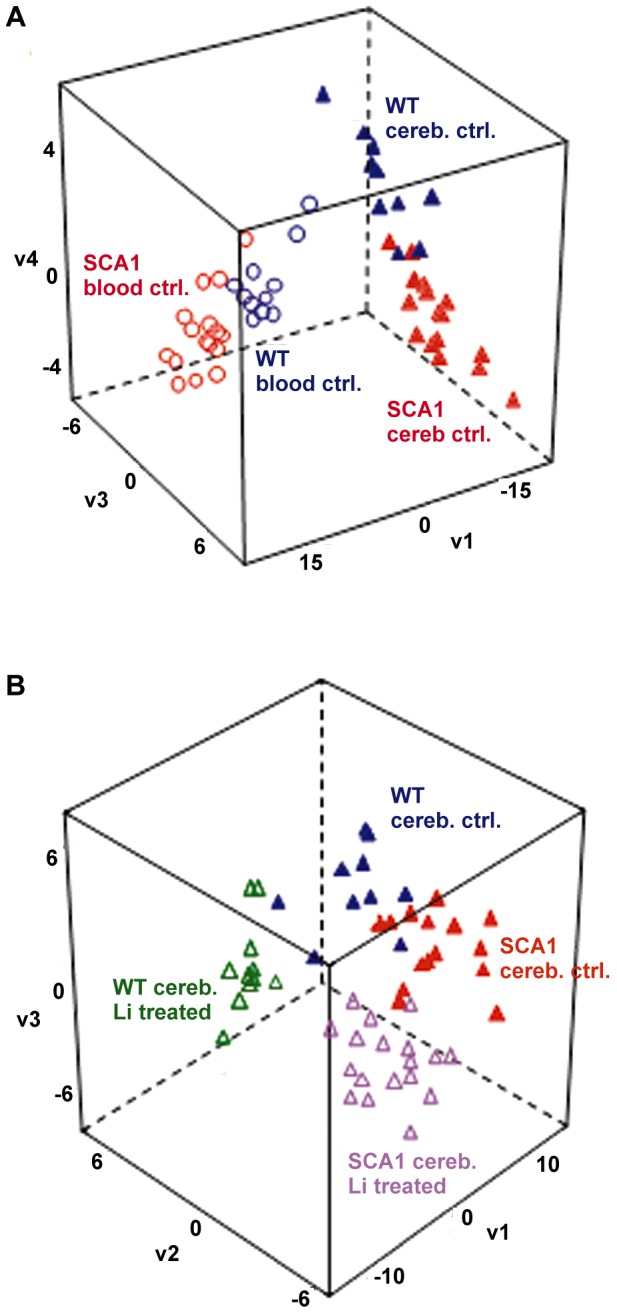
Supervised multivariate Partial Least Square separation of metabolic phenotypes. **A.** Differences between plasma and cerebellum (vector v1) and between SCA1 and wild-type under control conditions (separated by vectors v3 and v4). **B.** Differences between Lithium treated and control conditions in cerebellum (vector v1) and between SCA1 and wild-type (separated by vectors v2 and v3). Abbreviations: Cereb, Cerebellum; Ctrl, Control; Li, Lithium; WT, Wild-type.

We compared metabolic profiles of cerebellum and blood in *Sca1^154Q/+^* and wild-type mice. Figure 1A shows good separation of the four groups (plasma wild-type, plasma *Sca1^154Q/+^*, cerebellum wild-type and cerebellum *Sca1^154Q/+^*). The overall variance was clearly dominated by the differences in metabolite levels between plasma and cerebellar tissue, indicated by the contribution of vector 1 (48% of the overall metabolic variance). Differences that separated the two genotypes in both organs (vector 3 and 4) used only 5% of the overall metabolic variance, while vector 2 (not shown) was related to within-group variance. The clear separation of metabolic clusters in both blood plasma and cerebellum demonstrated that the disease-causing mutation is responsible for metabolic differences that underlie the disease phenotype in a systemic manner and were not confined to the brain. Using one-way ANOVA tests for each comparison, 41 metabolites were found to be differentially regulated at *p*<0.05 between wild-type and *Sca1^154Q/+^* mice in cerebellum, out of which 18 were structurally identified using authentic standards.

**Figure 2:**
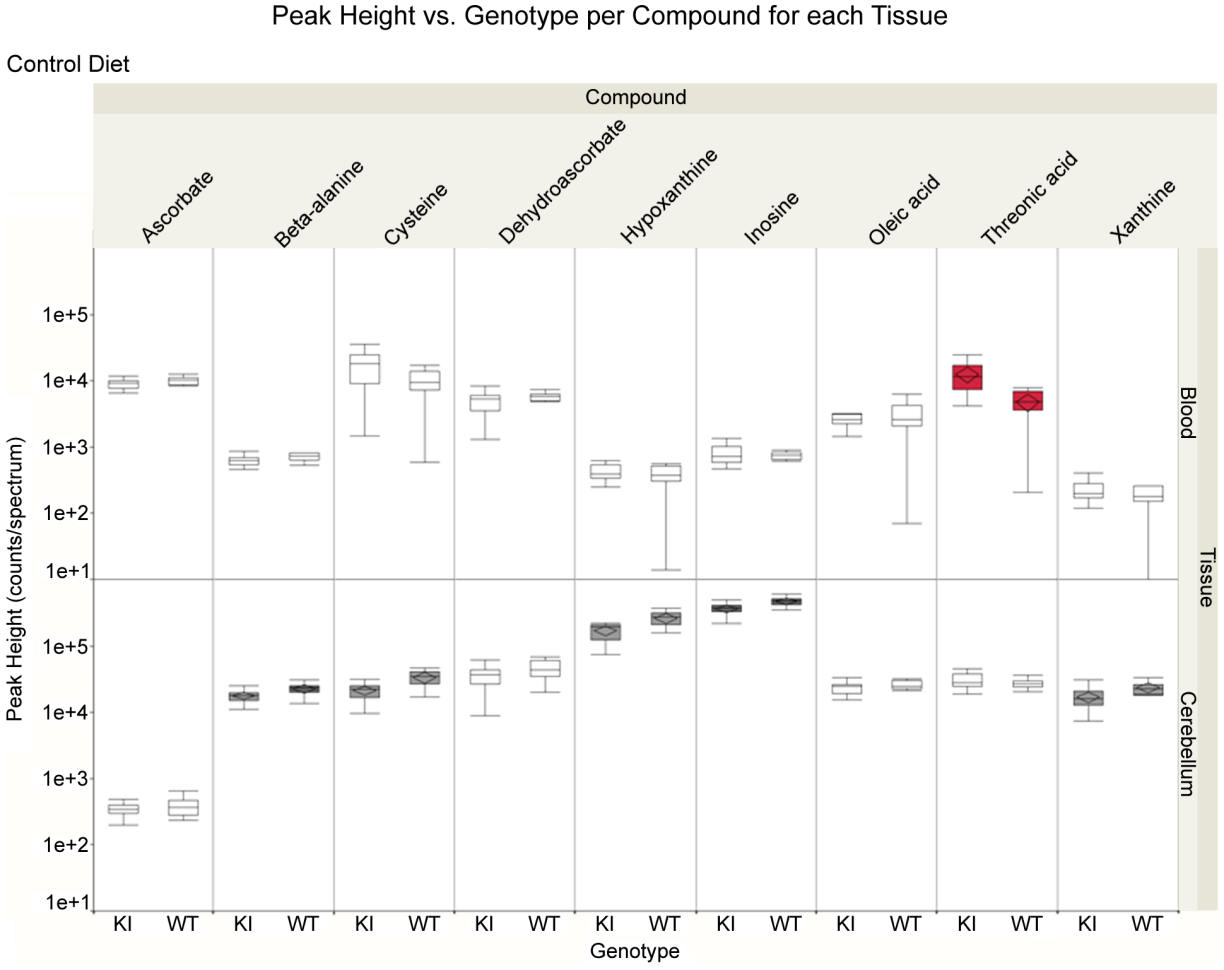
Effect of introducing the *Sca1^154Q/+^* gene into the wild-type genetic background for plasma and cerebellum. Individual box-whisker plots for selected significantly regulated metabolites. The whiskers encompass 1.5 of the interquartile range (IQR). Median value is indicated with a line in the box. The confidence diamonds indicate average values when the two samples are statistically different (colored boxplots, red for blood and grey for brain). Abbreviations: KI, SCA1 knock-in; WT, Wild-type.

We next set out to define the metabolic changes induced by polyglutamine (polyQ) expansion in *Sca1^154Q/+^* mice in plasma and cerebellum. Table 2 shows all compounds found in plasma or cerebellum at *p*<0.05 with the magnitude of difference in metabolite levels, and shows the ratios of metabolites in *Sca1^154Q/+^*/wild-type that differ between these two groups. A wide range of metabolites were down-regulated in *Sca1^154Q/+^* mice and few were up-regulated. Metabolites that changed in the cerebellum included intermediates in glucose, lipid, tricarboxylic acid (TCA) cycle and purine metabolism, as well as a number of amino acids including cysteine, beta-alanine and serine. The plasma of *Sca1^154Q/+^* mice showed higher levels of tryptophan and threonic acid, and lower levels of phosphate and amino adipic acid (Table 2). None of the identified metabolites were significantly different in both organs, which points to specific impact of the polyQ-expanded ATXN1 on both cerebellar biochemistry and whole-body physiology. The difference in metabolite levels between organs and the specific impact of the SCA1 gene on plasma and cerebellum metabolism is visualized for nine selected metabolites in Figure 2 (see Figure S1 for box-whisker plots of other significant compounds from Table 2).

**Table 2:** Genotype effect on metabolic profiles: Significantly different metabolites comparing wild-type versus *Sca1^154Q/+^* mice under control conditions.

Compounds	Average level SCA1/Wild-Type		p-value	
	Cerebellum	Plasma	Cerebellum	Plasma
Glucose-6-phosphate	0.5	1.1	**0.032**	0.614
Cysteine	0.6	1.6	**0.0004**	0.057
Hypoxanthine	0.6	1.3	**0.0004**	0.291
Phosphoric acid	0.7	0.9	**0.019**	0.270
Beta-Mannosylglycerate	0.7	0.9	**0.014**	0.647
Phosphate	0.7	0.7	0.145	**0.047**
2-monopalmitin	0.7	1.0	**0.005**	0.790
Xanthine	0.7	1.1	**0.007**	0.873
Galactinol	0.7	2.0	**0.003**	0.361
Citric acid	0.8	1.1	**0.030**	0.378
Monopalmitin-1-glyceride	0.8	0.9	**0.030**	0.542
Hydroxycarbamate NIST	0.8	1.4	**0.021**	0.088
Inosine	0.8	1.2	**0.003**	0.241
Beta-alanine	0.8	0.6	**0.012**	0.136
2-aminoadipic acid	0.8	0.6	0.137	**0.015**
Serine	0.8	1.1	**0.010**	0.353
Glycerol-3-galactoside	0.8	1.5	**0.014**	0.372
Inositol allo-	0.8	0.8	**0.043**	0.225
Tryptophan	1.0	1.5	0.840	**0.012**
Idonic acid NIST	1.1	2.5	0.426	**0.001**
Threonic acid	1.2	2.6	0.209	**0.001**
Glycolic acid	1.2	0.9	**0.028**	0.414
Hexuronic acid	1.2	1.0	**0.017**	0.839

Notes: Bold indicates statistical significance. One-way analysis of variance performed separately for cerebellum and blood plasma (see Figure 2 and supplemental Figure 1 for box-whisker plots). Abbreviation: NIST, National Institute of Standards and Technology.

### Comparison of central and peripheral biochemical changes caused by lithium treatment

We investigated the effect of lithium treatment for both genotypes, investigating individually for each organ. Eighty-five metabolites significantly differed under lithium treatment (*p*<0.05, one-way ANOVA) in at least one of the genotypes. Forty-two of these compounds were structurally identified and are listed in Table 3 with their respective *p*-values and magnitude of alteration. In cerebellar tissue, lithium treatment caused the largest impact on metabolic variation, affecting 42 of 44 significantly-affected compounds. This led us to conclude that gender, weight and age (other factors evaluated) of the mice were not relevant parameters in this study.

**Table 3:** Effect of Lithium treatment on cerebellum metabolic profile: Significantly different metabolites comparing Lithium treatment versus control conditions.

Compound	Average Level Li/control		p-value	
	SCA1	Wild-Type	SCA1	Wild-Type
Ascorbic acid	1.0	10.6	0.609	**0.002**
Glucose-6-phosphate	4.3	3.7	**0.000**	**0.010**
Fructose-6-phosphate	2.7	2.8	**0.000**	**0.007**
Elaidic acid	2.2	2.3	**0.001**	**0.009**
Glucose	1.8	2.3	**0.017**	**0.021**
Hypoxanthine	2.3	2.2	**0.000**	**0.000**
Fructose	1.8	1.6	0.118	**0.001**
Methionine	1.5	1.6	**0.000**	**0.000**
Maltose	2.9	1.6	**0.000**	**0.019**
Cysteine	1.8	1.6	**0.000**	**0.000**
Xanthine	1.8	1.5	**0.000**	**0.003**
Inositol-4-monophosphate	1.0	1.5	0.952	**0.015**
Citric acid	1.8	1.4	**0.000**	**0.001**
Tyrosine	1.2	1.4	**0.041**	**0.023**
Inosine	1.5	1.4	**0.000**	**0.001**
Fumaric acid	1.1	1.3	0.509	**0.009**
Oleic acid	1.2	1.3	**0.001**	**0.008**
Isocitric acid	1.5	1.3	**0.000**	**0.004**
Icosenoic acid	1.3	1.3	**0.038**	0.109
Linoleic acid	1.2	1.3	**0.033**	**0.010**
Tryptophan	1.3	1.3	**0.015**	**0.039**
Malic acid	1.2	1.2	**0.027**	**0.008**
Phenylalanine	1.2	1.2	**0.020**	**0.019**
Pseudo uridine	1.2	1.2	**0.009**	**0.016**
Pantothenic acid	1.3	1.2	**0.006**	0.087
Valine	1.2	1.2	0.134	**0.017**
Ornithine	1.3	1.2	**0.009**	0.132
Glycine	1.1	1.1	0.166	**0.037**
Monopalmitin-1-glyceride	1.3	1.1	**0.008**	0.300
Methionine sulfoxide	1.3	1.1	**0.007**	0.470
2-aminoadipic acid	1.3	1.0	**0.043**	0.799
Glycerol-alpha-phosphate	0.7	1.0	**0.043**	0.907
2-monopalmitin	1.4	1.0	**0.003**	0.698
Alloxanoic Acid NIST	0.7	0.9	**0.001**	0.324
Ethanolamine	0.9	0.8	0.475	**0.032**
Ribonic acid	0.9	0.8	0.400	**0.030**
Theonic acid	1.0	0.8	0.948	**0.007**
Nicotinamide	0.9	0.8	0.512	**0.042**
Glyceric acid	1.0	0.8	0.813	**0.007**
Phytol	1.1	0.6	0.664	**0.002**
Adenosine-5-monophosphate	0.5	0.5	**0.001**	**0.011**
Cholestan-3-ol	0.9	0.5	0.566	**0.015**

Note: Bold indicates statistical significance. One-way ANOVA performed separately for wild-type and *Sca1^154Q/+^* mice (see Figure 5 and supplemental Figure 3 for box-whisker plots). Abbreviation: Li, Lithium.

We also found lithium treatment to be a significant parameter for separating all cerebellar samples using PLS analysis (Figure 1B) with vector 1 explaining 23% of the group-dependent variance. Vectors 2 and 3 were not significant by themselves, but taken together were able to classify the distinct effects of the wild-type and *Sca1^154Q/+^* mice under lithium treatment and control conditions (explaining 29% of the group-dependent metabolic variance). While even unsupervised multivariate analysis (principal components analysis) could readily distinguish samples between lithium-treated and untreated animals (graph not shown), the within-group variance was too high to distinguish the effects of *Sca1^154Q/+^* without the classification power of supervised PLS.

To pinpoint the significance level for each metabolite individually, we used univariate statistics to compare lithium-treated versus control conditions in wild-type and in *Sca1^154Q/+^* mice—separately for both—in the cerebellum and plasma (see below). Using multivariate ANOVA and using metabolites as dependent variables and study parameters as covariates (lithium, genotype, sex, age and weight), we found only 20 metabolites to be significantly impacted by at least one parameter. Gender was found to be significant for isoleucine, allo-inositol and aminoadipic acid. As these metabolites were not significantly different per lithium treatment, subsequent statistical analyses were performed by combining data from both sexes. Using one-way ANOVA tests, 59 plasma metabolites were significantly differentially regulated under lithium treatment when comparing both genotypes (wild-type and *Sca1^154Q/+^*; ANOVA one-way p<0.05). Twenty-eight of these compounds were annotated with identified structures (Table 4; see Figure S2 for box-whisker plots of significant compounds).

**Table 4:** Effect of Lithium treatment on blood plasma metabolic profile: Significantly different metabolites comparing Lithium treatment versus control conditions.

Compound	Average Level Li/control		p-value	
	SCA1	Wild-Type	SCA1	Wild-Type
Icosenoic acid	2.0	2.1	**0.042**	0.287
Malic acid	1.8	1.8	**0.000**	**0.001**
2-aminoadipic acid	1.7	1.1	**0.000**	0.532
Fumaric acid	1.5	1.5	**0.009**	**0.018**
Glyceric acid	1.4	1.1	**0.005**	0.314
Phosphate	1.3	0.9	**0.030**	0.464
Dehydroascorbate	1.3	2.2	**0.014**	0.283
Ascorbic acid	1.3	1.1	**0.021**	0.407
Aconitic acid	1.3	1.7	0.115	**0.015**
Glycocyamine	1.3	3.7	**0.014**	0.254
Citric acid	1.3	1.5	**0.002**	**0.000**
Methionine	1.2	1.4	0.220	**0.028**
Isocitric acid	1.2	1.3	**0.039**	0.054
Stearic acid	1.2	1.6	0.092	**0.002**
Pseudo uridine	1.2	1.4	**0.044**	**0.003**
Indole-3-lactate	1.1	1.5	0.164	**0.000**
Palmitic acid	1.1	1.4	0.272	**0.006**
Indole-3-acetate	1.1	1.5	0.472	**0.006**
N-acetylaspartic acid	0.9	1.3	0.623	**0.007**
Sucrose	0.9	1.6	0.713	**0.019**
Aminomelonic acid	0.9	1.6	0.435	**0.001**
Hydroxycarbamate	0.9	1.5	0.338	**0.016**
Oxoproline	0.8	1.0	**0.004**	0.739
1-monostearin	0.8	0.9	**0.023**	0.738
Pyrazine 2,5-dihydroxy	0.7	1.1	**0.025**	0.544
Cysteine	0.6	1.1	**0.006**	0.663
Idonic acid NIST	0.5	1.2	**0.000**	0.671
Threonic acid	0.4	1.2	**0.000**	0.429

Note: Bold indicates statistical significance. One-way ANOVA performed separately for wild-type and *Sca1^154Q/+^* mice (see supplemental Figure 2 for box-whisker plots). Abbreviation: Li, Lithium.

### Plasma and cerebellar metabolic signature for lithium treatment in wild-type mice

Lithium treatment in wild-type mice led to significant changes in 33 metabolites in cerebellum and 14 in plasma. Malic acid, fumaric acid, citric acid, methionine and pseudo uridine showed changes in both tissues.

**Figure 3:**
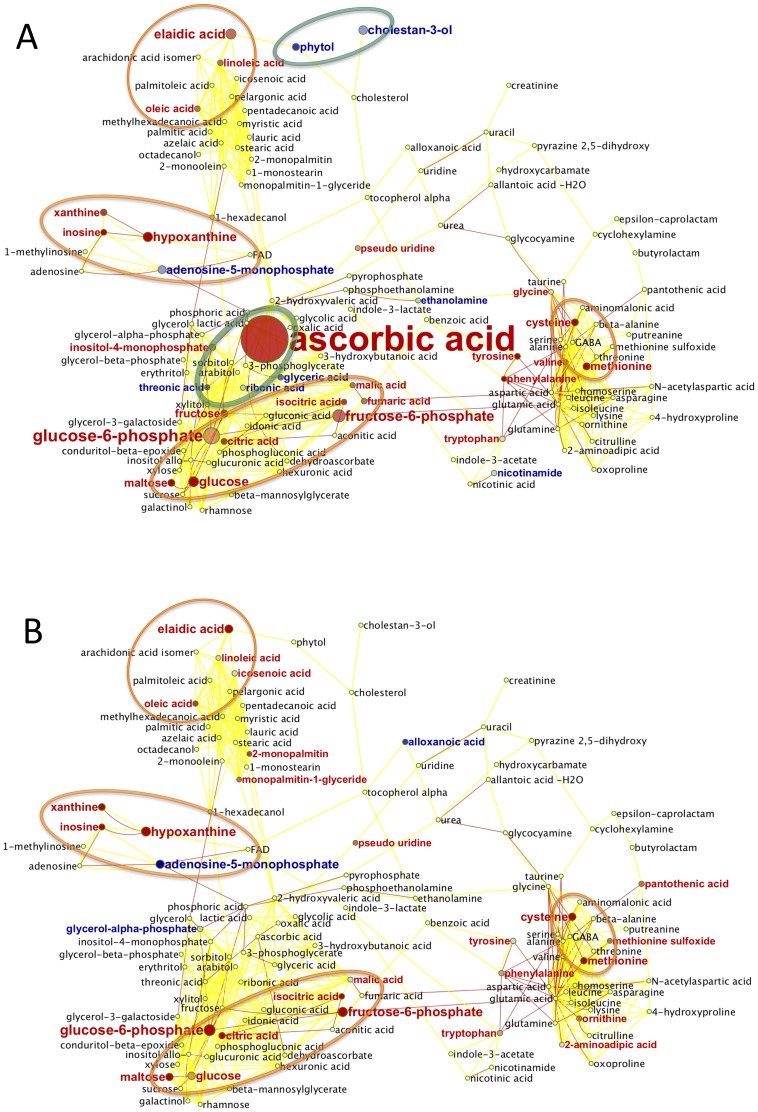
Effect of lithium treatment on cerebellum metabolome. Metabolic network of wild-type and *Sca1^154Q/+^* cerebellum phenotypes. **A.** Wild-type mice. **B.** SCA1 knock-in mice. Red nodes: Increased metabolite levels under Lithium treatment; blue nodes: decreased levels. Node shades indicate ANOVA significance levels, node size reflect differences in magnitude of regulation. Red lines: reactant pair relationships obtained from the KEGG reaction pair database. Yellow solid lines: chemical similarity >0.5 Tanimoto score (Tanimoto scores range between 0 to 1, where 1 reflects identical structures). Yellow broken lines: chemically closest structure at <0.5 Tanimoto scores. Green circles group significant compounds that changed only in the Wild-type genotype. Orange circles group significant compounds that changed in both genotypes.

Lithium effects seen in wild-type mice (Table 3 and Figure 3 panel B, orange circles) were clustered to the purine biochemical pathway (increased levels of xanthine, inosine, hypoxanthine), sulphur and aromatic amino acids (increased levels of cysteine, methionine, tryptophan, phenylalanine) and central energy metabolism (increased concentrations for fructose, glucose, fructose-6-phosphate, glucose-6-phosphate and TCA metabolites citrate, isocitrate, malate and fumarate with concomitant decreased levels of adenosine monophosphate [AMP]). There was also a dramatically increased level of ascorbate which was accompanied by reduction of threonate, and slightly increased levels of unsaturated free fatty acids (elaidic acid, oleic acid and linoleic acid).

**Figure 4:**
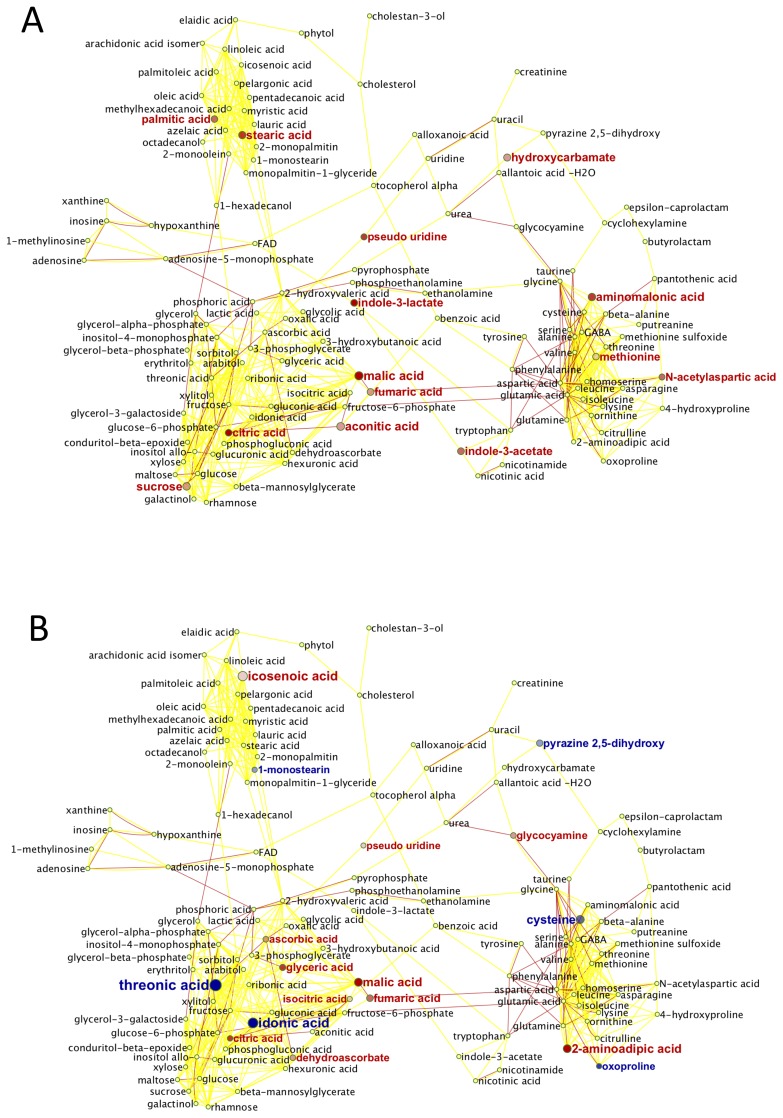
Lithium treatment effect on blood plasma metabolome. Metabolic network of wild-type and *Sca1^154Q/+^* plasma phenotypes. **A.** Wild-type mice. **B.** SCA1 knock-in mice. Red nodes: Increased metabolite levels under Lithium treatment; blue nodes: decreased levels. Node shades indicate ANOVA significance levels, node size reflect differences in magnitude of regulation. Red lines: reactant pair relationships obtained from the KEGG reaction pair database. Yellow solid lines: chemical similarity >0.5 Tanimoto score (Tanimoto scores range between 0 to 1, where 1 reflects identical structures). Yellow broken lines: chemically closest structure at <0.5 Tanimoto scores.

While lithium treatment was the most significant study parameter affecting metabolite levels in plasma, it had only moderate effects on the identified metabolites (Figure 4). Similar to what was found in cerebellar tissues, TCA metabolites were increased with lithium treatment in wild-type mice (citric, aconitic, isocitric, malic and fumaric acid), albeit at low abundance differences.

### Metabolic signature and therapeutic effect of lithium treatment in *Sca1^154q/+^* mice

**Figure 5:**
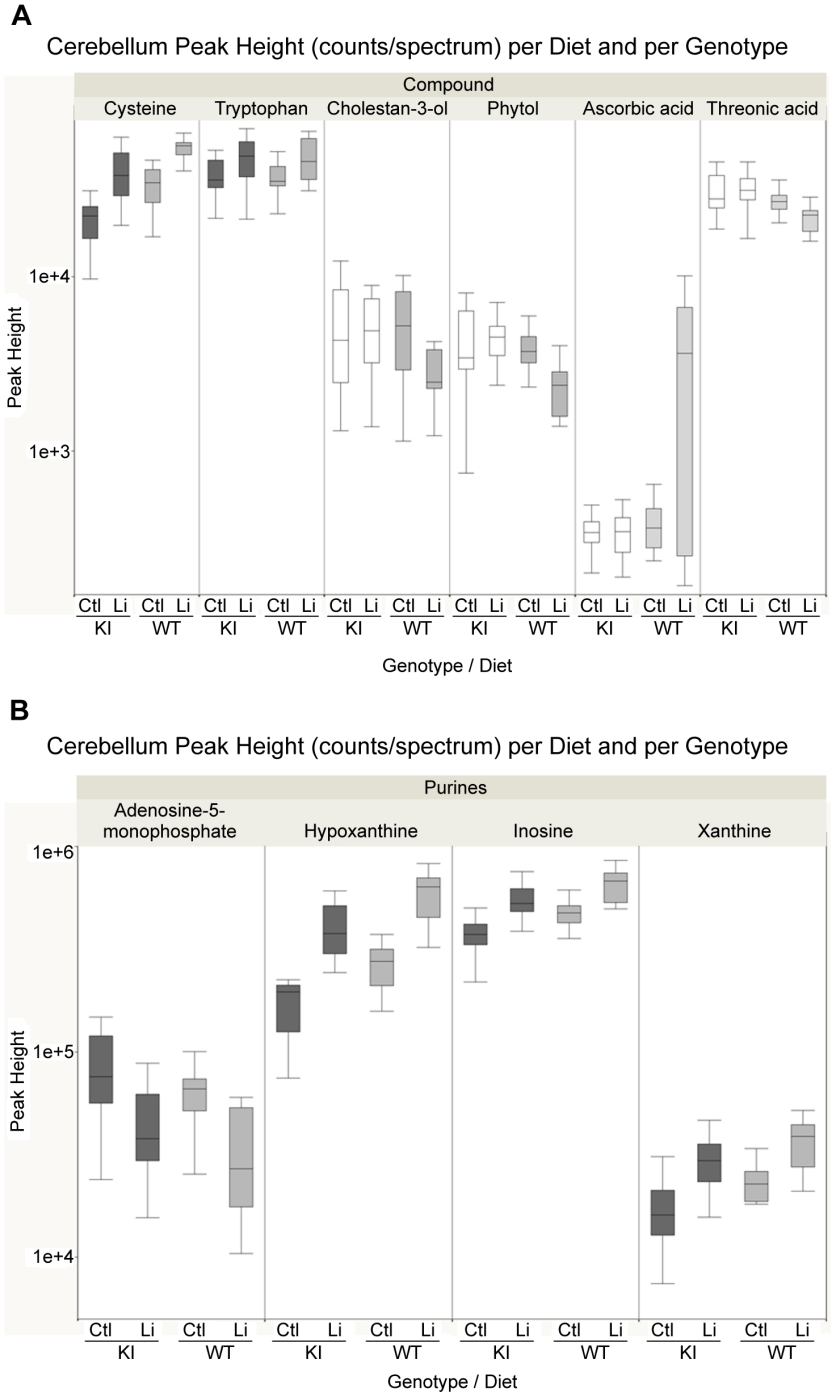
Box-and-whisker plots: genotype-dependent metabolites in cerebellum tissue with significant differences between lithium and controls (*p*<0.05). **A.** Box-whisker plots for selected significantly regulated metabolites. **B.** Box-whisker plots for significantly regulated metabolites of purine metabolism pathway. The whiskers encompass 1.5 of the interquartile range (IQR). Median value is indicated with a line in the box. Boxes are filled in color (dark grey: SCA1 knock-in; light grey: wild-type) when the samples are statistically different between the two lithium treatments. Abbreviations: Ctl, Control; KI, SCA1 knock-in; Li, Lithium; WT, Wild-type.

Of 29 significantly altered metabolites in *Sca1^154Q/+^* mouse cerebellum after lithium treatment, nine were specific to *Sca1^154Q/+^* mice. Moreover, lithium treatment affected 12 metabolites in wild-type mice only. Those compounds that only responded to lithium in one of the genotypes (Table 3 and Figure 3 Panel A, green circles) indicate metabolic pathways that might be disturbed by the action of the mutant *Atxn1*. For example, ascorbic acid (Vitamin C) exhibited the overall highest fold change in the cerebellum of wild-type mice (10-fold accumulation, one-way ANOVA p = 0.002) but not *Sca1^154Q/+^* mice. The increase of ascorbate in wild-type mice was accompanied by a reduction of threonate (Figure 3 and Figure 5), which is an oxidative catabolite of ascorbate (reaction pair KEGG RP01024). Interestingly, the threonic acid/ascorbate reaction pair and dehydroascorbate were differentially regulated in the plasma of *Sca1^154Q/+^* mice but not wild-type mice, which was opposite to the finding in cerebellum. In a different metabolite module, phytol and cholestan-3-ol were also affected by lithium treatment in wild-type mice but not in *Sca1^154Q/+^* mice, albeit with a lower p-value (Table 3). Box-whisker graphs for selected metabolites (Figure 5) highlight common and specific responses to lithium treatment in the cerebellum (see Figure S3 for box-whisker plots of other compounds from Table 3).

To assess the therapeutic effects of lithium, we looked at the altered metabolites in *Sca1^154Q/+^* mice that were corrected by lithium treatment (Table 5). In the plasma of *Sca1^154Q/+^* mice, the levels of phosphate and 2-aminoadipic acid were down and the levels of idonic acid NIST (National Institute of Standards and Technology), threonic acid and tryptophan were up compared to the wild-type control animals. All of these, except for tryptophan, were restored to their normal levels by lithium treatment. Interestingly, none of these compounds were affected by lithium treatment in wild-type mice. Therefore, these four metabolites might be indicators of the disease suppression.

**Table 5:** Level of metabolites in SCA1 mice relative to wild-type non-treated mice: the treatment effect of lithium on impaired metabolites in SCA1 knock-in mice.

Compounds	Wild-type		*Sca1^154Q/+^*	
	control	lithium	control	lithium
	Avg±SD	Avg±SD	Avg±SD	Avg±SD
Cerebellum				
Glucose-6-phosphate	1.0±0.9	3.8±2.9	0.5±0.2	2.0±1.3
Cystein	1.0±0.3	1.6±0.3	0.7±0.2	1.2±0.4
Hypoxanthine	1.0±0.2	2.2±0.6	0.7±0.2	1.5±0.5
2-monopalmitin	1.0±0.3	1.0±0.3	0.7±0.2	1.0±0.3
Xanthine	1.0±0.2	1.5±0.4	0.7±0.2	1.3±0.4
Citric acid	1.0±0.2	1.4±0.3	0.8±0.3	1.4±0.3
Monopalmitin-1-glyceride	1.0±0.2	1.1±0.3	0.8±0.3	1.0±0.2
Inosine	1.0±0.2	1.4±0.3	0.8±0.2	1.2±0.2
Blood Plasma				
Phosphate	1.0±0.3	1.0±0.1	0.9±0.1	1.0±0.2
2-aminoadipic acid	1.0±0.5	1.1±0.4	0.6±0.3	1.0±0.3
Tryptophan	1.0±0.5	1.2±0.3	1.5±0.5	1.3±0.5
Idonic acid NIST	1.0±1.0	1.2±0.7	2.5±1.1	1.2±0.9
Threonic acid	1.0±0.4	1.2±0.9	2.6±1.3	1.1±0.6

Note: All values are relative to wild-type untreated animals. Abbreviations: Avg, Average; NIST, National Institute of Standards and Technology; SD, Standard deviation.

Similarly in the cerebellum, the levels of two closely-related compounds, 2-monopalmitin and monopalmitin-1-glyceride, were restored by lithium treatment in *Sca1^154Q/+^* mice but were not elevated in the wild-type lithium-treated animals. Among the affected metabolites in the cerebellum of the *Sca1^154Q/+^* mice, six compounds were also affected by lithium treatment. The level of these metabolites decreased in *Sca1^154Q/+^* mouse cerebellum and they were increased upon lithium treatment regardless of genotype. In particular, when checking the ratio of hypoxanthine and its corresponding nucleoside, inosine is similar in both the wild-type and *Sca1^154Q/+^* mice, respectively 0.55±0.07 (average ± standard deviation) and 0.47±0.09. Upon lithium treatment, we observed an increase in the hypoxanthine/inosine ratio by 56% to 0.86±0.15 for the wild-type and 54% to 0.72±0.13 for the *Sca1^154Q/+^* mice. Concurrent to that relative increase of hypoxanthine, the level of adenosine monophosphate is reduced by the lithium treatment for both genotypes (Figure 5B).

## Discussion

This study used a non-targeted metabolomics approach to map global changes in metabolism centrally and peripherally in a mouse model of ataxia before and after treatment with lithium. Pure metabolic differences were noted in the plasma of *Sca1^154Q/+^* mice compared to wild-type mice, which underscores the importance of profiling relevant tissue in neurological disorders. In plasma, threonate was significantly reduced in the *Sca1^154Q/+^* mice, but neither ascorbate nor dehydroascorbate were significantly different in both cerebellum and plasma. The amino acids beta-alanine and cysteine were significantly decreased in cerebellum, as were the purines xanthine, hypoxanthine and inosine.

Lithium neuroprotection mechanism is unclear, even if stimulation of anti-apoptotic pathways has been shown, inhibiting the activity of a serine/threonine kinase, the glycogen synthase kinase 3β (GSK3β) [25]. Lithium treatment also affects glucose transport, enhances energy metabolism, particularly glucose metabolism [26]–[28]. Corroborating with this notion our data show that impaired central energy metabolism was restored to some degree by lithium treatment.

The pathways affected by lithium—including the purine biochemical pathway, sulphur-containing and aromatic amino acids, central energy metabolism, unsaturated free fatty acids, ascorbate and threonate—might all be related to the disease or to benefit from treatment.

Threonate is a product of ascorbate oxidative cleavage (KEGG reaction pair RP01024). The changes in ascorbate and threonate only happened in lithium-treated wild-type but not *Sca1^154Q/+^* mice. While we have not measured ascorbate levels in the diet, both genotypes were fed the same Purina rodent chow with or without the lithium supplement. Therefore, the observed differences cannot be related to how much ascorbate was provided. As these differences were not seen in the plasma profiles of wild-type mice, it is possible that lithium-treated wild-type mice respond by a reduced catabolism of the antioxidant ascorbate into threonate in the cerebellum (enzyme EC 1.13.11.13). This lithium response appears to be suppressed in the *Sca1^154Q/+^* mice. Ascorbate can alternatively be oxidized to dehydroascorbate (enzyme EC 1.10.3.3) [29]. The *Sca1^154Q/+^* mice showed differential levels of threonate, ascorbate and dehydroascorbate in blood plasma, but not in the cerebellum. The finding that the levels of ascorbic acid and dehydroascorbate were much higher in blood plasma than in cerebellum tissue suggests that lithium treatment might activate ascorbate/dehydroascorbate uptake and is at least partly involved in the ascorbate accumulation in the cerebellum. Targeted experiments are required to further elucidate this complex response.

Another pair of metabolites that respond differentially to lithium treatment in cerebellar tissue between wild-type and *Sca1^154Q/+^* mice is phytol and cholestan-3-ol (dihydrocholesterol). Cholestan-3-ol was decreased in the cerebellum of lithium-treated wild-type mice, but not in the cerebellum of lithium-treated *Sca1^154Q/+^* mice. Cholestan-3-ol binds to NCP2, which is associated with the Nieman-Pick C2 neurodegenerative disease [30]. Deposits of cholestan-3-ol in the brain define the hallmark of Cerebrotendinous xanthomatosis, a rare genetic disease that is characterized by progressive cerebellar ataxia [31]–[33]. The effect of lithium on reducing levels of this cholesterol derivative in the cerebella of wild-type but not *Sca1^154Q/+^* mice indicates that the mechanisms that control the levels of this metabolite are impaired in SCA1. Significant accumulation of ascorbate in the cerebella of lithium-treated wild-type but not SCA1 mice, and altered levels of phytol (precursor for antioxidant Vitamin E biosynthesis) and cholestan-3-ol (a similar compound exhibiting the same response to lithium treatment) suggest that part of the lithium neuroprotective properties could be mediated by reducing oxidative stress.

Inhibitory effects of lithium on components of inositol phosphate metabolism have been suggested before [7]. Lithium is an uncompetitive inhibitor of inositol phosphate metabolism and the chronic lithium treatment leads to accumulation of inositol monophosphates (InsP) [34]. Our result shows an increase of inositol-4-monophosphate (Ins4P) upon lithium treatment, however this only happens in the wild-type but not in the mutant animals.

The observed increase in aromatic and sulfur-containing metabolites has been previously reported as an effect of lithium treatment for the regulation of sulfur metabolism and cysteine-string proteins in rat brain [35], [36]. Sulfur amino acids have already been associated with neurodegenerative disease [37].

Several of the pathways differentially regulated by the lithium treatment in both wild-type and *Sca1^154Q/+^* mice are well known to be important for neurological diseases. The nucleotide salvage pathway allows the cell to produce nucleotide monophosphates as the de novo synthesis pathway is non-existent in the brain. Measurement of AMP in the brain is dependent upon the sample preparation as adenosine triphosphate can degrade to adenosine diphosphate and AMP. The similar and statistically significant responses observed in both genotypes indicate that the sample preparation was proper and homogenous. Lithium treatment causes a ≥50% increase in the efficiency of the purine salvage pathway that converts inosine into hypoxanthine in both the SCA1 and wild-type genotypes in a very similar way. The differential regulation of the purines xanthine, hypoxanthine, inosine and AMP under lithium treatment is consistent with the hypothesis of a central role for this pathway in depression [38]. Xanthosine, the corresponding nucleoside of xanthine, has been linked to stagger in sheep, thus associating purine to motor neuron disease [39]. Further, recent studies have shown that ascorbate levels act on the purine metabolism pathway, which suggests that the effect of lithium on ascorbate and purine metabolites that was observed in this study could be linked [40].

Neurodegenerative disorders have also been reported to exhibit the loss of essential unsaturated fatty acids—such as linoleic acid—in membranes [41], and linoleic acid was increased after lithium treatment. Other neurodegenerative diseases have been associated with inefficient glycolysis. We found that lithium treatment increases the level of several molecules involved in glycolysis, which is a critical pathway for energy production in the cell. Lithium appears to boost levels of both glycolytic and TCA intermediates, and may therefore alleviate the reported inefficiency of glycolysis in neurological diseases [42]–[44].

None of these differential compounds appear directly associated with any of a list of 194 differential transcripts, per a micro-array analysis [45] performed on the same *Sca1^154Q/+^* mouse model, and similarly to the 19 differentials proteins of rat prefrontal cortex after lithium treatment [46].

In conclusion, our findings corroborate previous reports that lithium has multiple effects in neurological diseases. Our data further reveal that lithium affects metabolic intermediates in the cerebellum to a larger degree than in blood plasma. It also has a controlling role in a variety of metabolic pathways, including purine metabolism, oxidative stress reduction and energy production.

## Supporting Information

Figure S1 **Effect of introducing the** ***Sca1^154Q/+^*** **gene into the wild-type genetic background for plasma and cerebellum.** This figure includes individual box-whisker plots for other significantly regulated metabolites (i.e., compounds from Table 2 not shown in Figure 2). The whiskers encompass 1.5 of the interquartile range (IQR). Median value is indicated with a line in the box. Abbreviations: Ctl, Control; KI, SCA1 knock-in; Li, Lithium; WT, Wild-type.
(TIF)

Figure S2 **Box-and-whisker plots: genotype-dependent metabolites in plasma samples with significant differences between lithium treatment and controls.** (p-value <0.05; see Table 4). The whiskers encompass 1.5 of the interquartile range (IQR). Median value is indicated with a line in the box. Abbreviations: KI, SCA1 knock-in; WT, Wild-type.
(TIF)

Figure S3 **Box-and-whisker plots: genotype-dependent metabolites in cerebellum samples with significant differences between lithium treatment and controls.** (p-value <0.05; see Table 3). The whiskers encompass 1.5 of the interquartile range (IQR). Median value is indicated with a line in the box. Abbreviations: Ctl, Control; KI, SCA1 knock-in; Li, Lithium; WT, Wild-type.
(TIF)
