# Supplementary figures and images for: Pharmacometabolomic Signature of Ataxia SCA1 Mouse Model and Lithium Effects
Source: PLoS One. 2013 Aug 2;8(8):e70610. doi: 10.1371/journal.pone.0070610 (PMC3732229; doi:10.1371/journal.pone.0070610)

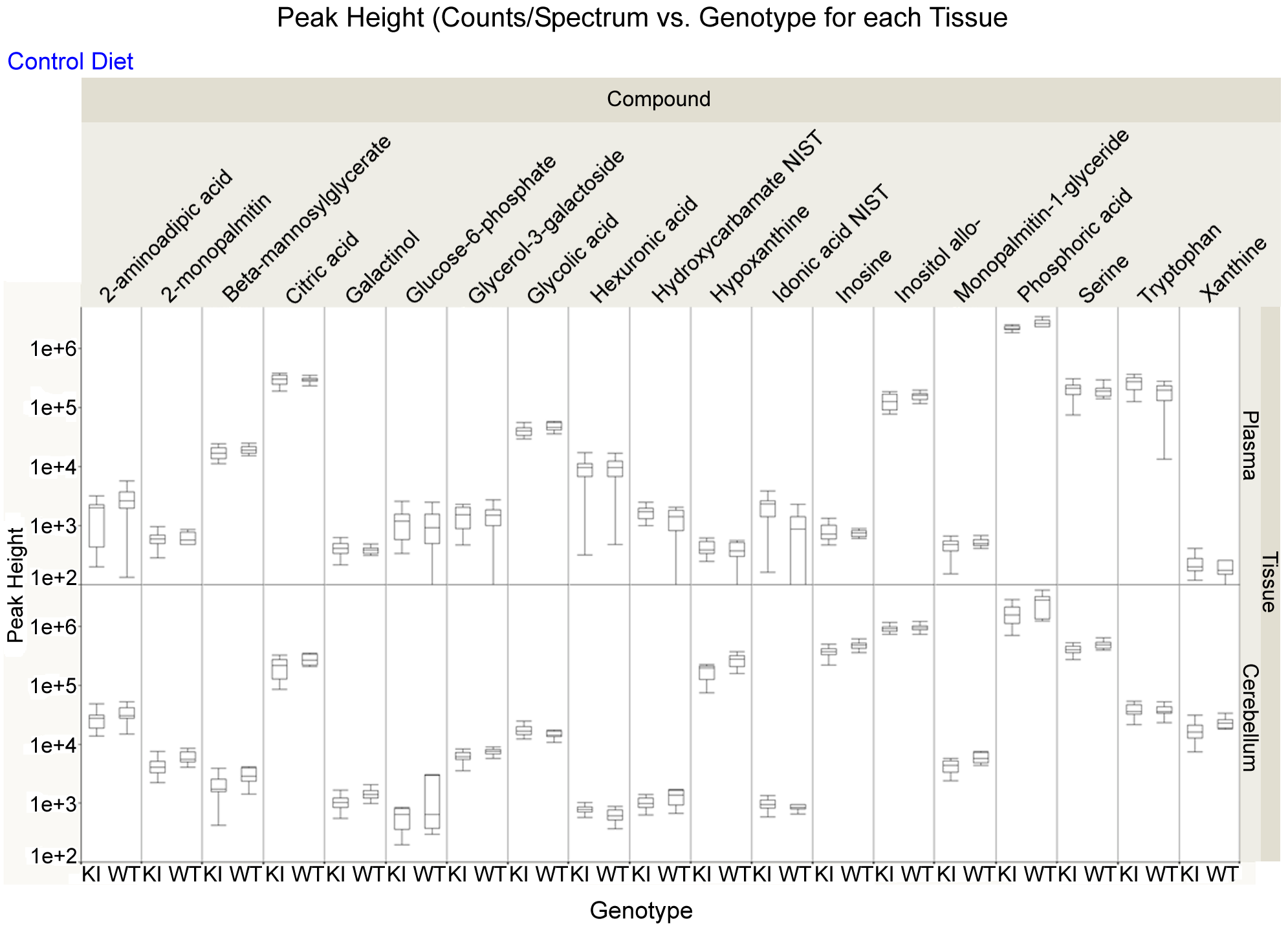

Supplement: Figure S1 — Effect of introducing the Sca1154Q/+ gene into the wild-type genetic background for plasma and cerebellum. This figure includes individual box-whisker plots for other significantly regulated metabolites (i.e., compounds from Table 2 not shown in Figure 2). The whiskers encompass 1.5 of the interquartile range (IQR). Median value is indicated with a line in the box. Abbreviations: Ctl, Control; KI, SCA1 knock-in; Li, Lithium; WT, Wild-type. (TIF) [file pone.0070610.s001.tif]

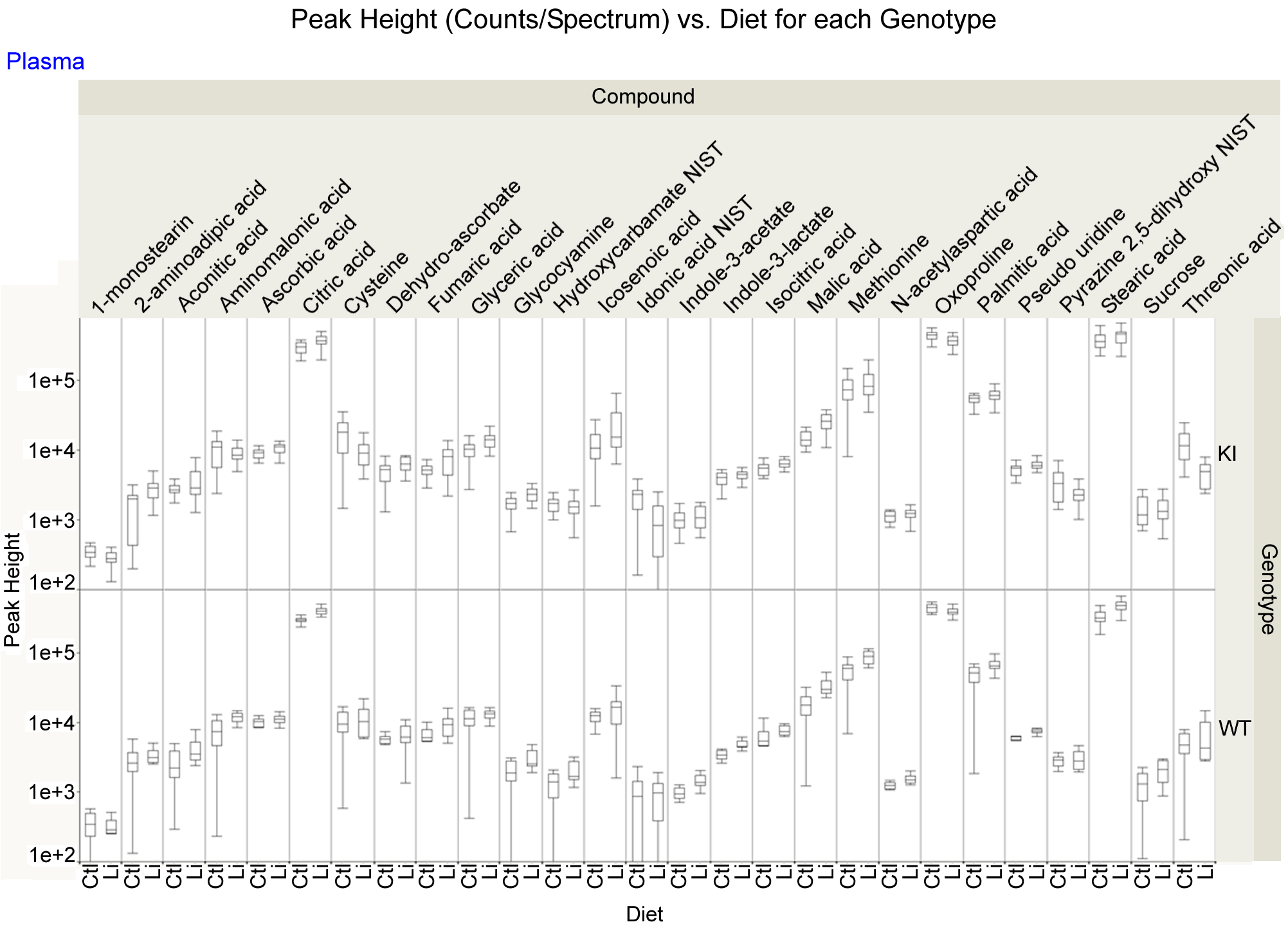

Supplement: Figure S2 — Box-and-whisker plots: genotype-dependent metabolites in plasma samples with significant differences between lithium treatment and controls. (p-value <0.05; see Table 4). The whiskers encompass 1.5 of the interquartile range (IQR). Median value is indicated with a line in the box. Abbreviations: KI, SCA1 knock-in; WT, Wild-type. (TIF) [file pone.0070610.s002.tif]

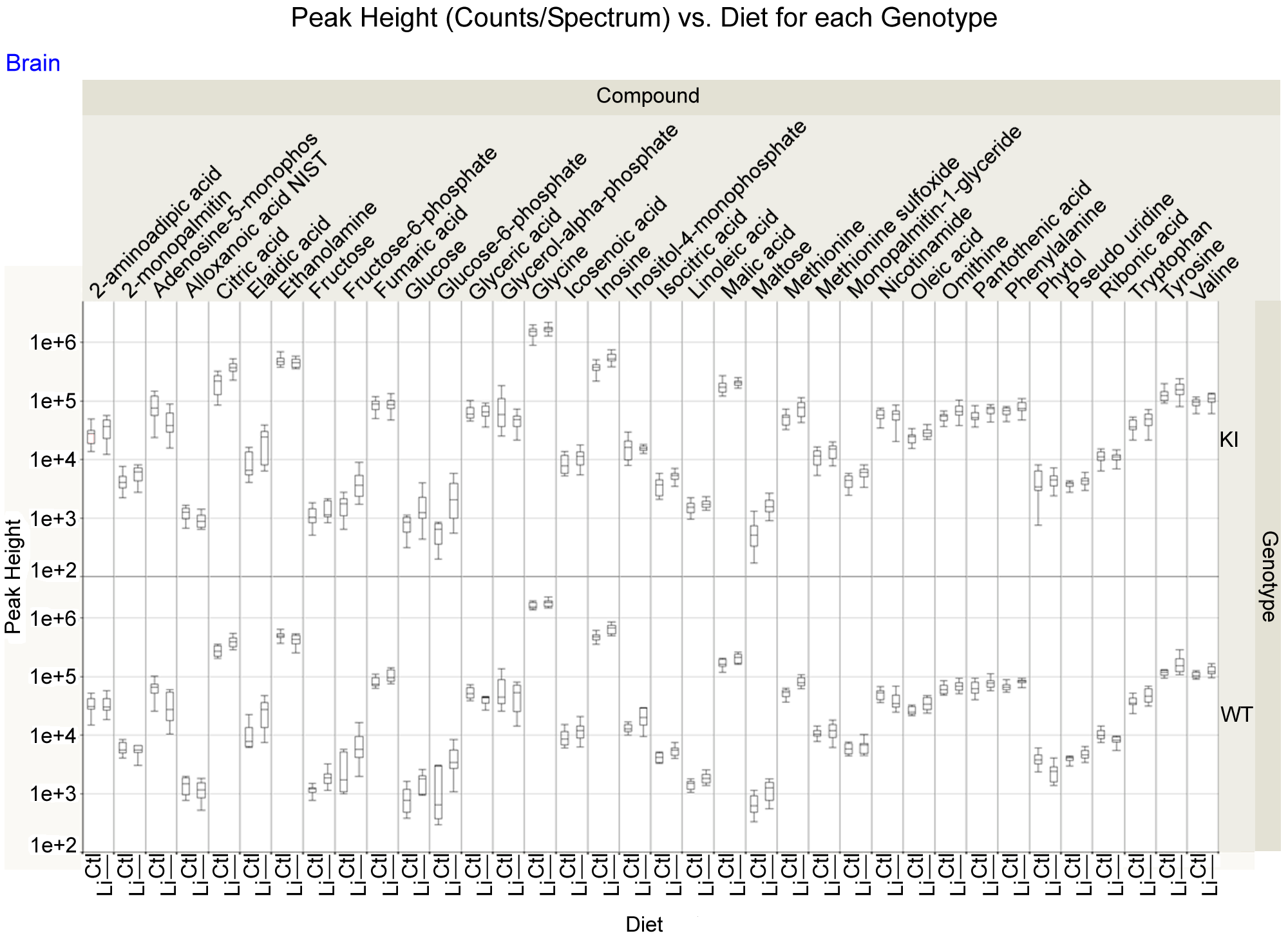

Supplement: Figure S3 — Box-and-whisker plots: genotype-dependent metabolites in cerebellum samples with significant differences between lithium treatment and controls. (p-value <0.05; see Table 3). The whiskers encompass 1.5 of the interquartile range (IQR). Median value is indicated with a line in the box. Abbreviations: Ctl, Control; KI, SCA1 knock-in; Li, Lithium; WT, Wild-type. (TIF) [file pone.0070610.s003.tif]
